# “PrevenganT2,” a Culturally Responsive Family-Based Diabetes Prevention Intervention for Hispanic or Latino Adults at High Risk for Type 2 Diabetes: Protocol for a Proof-of-Concept Evaluation

**DOI:** 10.2196/66317

**Published:** 2025-08-14

**Authors:** Brooke E Franklin, Brynn L Meulenberg, Elizabeth Z Beaulieu, Jessica Cisneros-Macias, Yiqing Cao, Sara Carbajal-Salisbury, Jeannette Villalta, Nelamaria Flores, Virginia Fuentes, Maria C Hernandez, Diana Parry-Alba, Carmen Rodriguez, Yolanda Rodriguez, Veronica S Zavala Orozco, Anu Asnaani, Ana Sanchez-Birkhead, Katherine J W Baucom

**Affiliations:** 1 Department of Psychology The University of Utah Salt Lake City, UT United States; 2 Alliance Community Services Salt Lake City, UT United States; 3 Community Advisory Board member Salt Lake City, UT United States; 4 College of Nursing The University of Utah Salt Lake City, UT United States

**Keywords:** community-based participatory research, cultural adaptation, family-based diabetes prevention, family support, health behavior change, Hispanic or Latino, lifestyle intervention, type 2 diabetes

## Abstract

**Background:**

Despite the efficacy of lifestyle interventions for preventing or delaying diabetes, community translations to date have failed to engage Hispanic or Latino participants effectively. Previously identified barriers to engagement include lack of family support and the burden of time-intensive year-long programs. Integrating family members and reducing program length may have the potential to increase engagement in lifestyle interventions to prevent type 2 diabetes in Hispanic or Latino individuals.

**Objective:**

Given the potential impact of cultural adaptation on such interventions, our community-academic research team used the Obesity-Related Behavioral Intervention Trials (ORBIT) model to guide an adaptation of the core 16 modules of the National Diabetes Prevention Program’s PrevengaT2 curriculum to meet the needs of Hispanic or Latino communities in Utah. We describe our evaluation of this adaptation in an ongoing proof-of-concept trial. We will evaluate whether Hispanic or Latino participants at high risk for type 2 diabetes increase their weekly moderate-to-vigorous physical activity (MVPA) in the context of an ongoing proof-of-concept trial of the adaptation.

**Methods:**

Target participants at risk for type 2 diabetes and a family member were invited to participate in PrevenganT2. In earlier phases of a larger project, our academic-community research team created the 14-week lifestyle intervention by adapting the Centers for Disease Control and Prevention’s PrevengaT2 curriculum. Objective MVPA was measured for 7 days at preintervention and postintervention using ActiGraph GT3X-BT accelerometers. Participants additionally completed questionnaires at preintervention and postintervention, and weight and self-reported MVPA were recorded at lifestyle intervention classes. Physical activity data will be analyzed to determine the percentage of target participants with clinically significant pre-post increases in MVPA.

**Results:**

Data collection concluded in October 2024. Data cleaning and preparation for analysis are ongoing. We expect that results will be submitted for publication by June 2025.

**Conclusions:**

This study serves as a first step in evaluating a novel, culturally adapted lifestyle intervention to prevent type 2 diabetes in Hispanic or Latino adults. Although this small study is not without limitations, findings will inform our team’s next steps for this early-phase intervention work.

**International Registered Report Identifier (IRRID):**

DERR1-10.2196/66317

## Introduction

Type 2 diabetes impacts an estimated 34-36 million Americans [1]. The rate of type 2 diabetes is expected to increase substantially in the coming decades and is increasingly common in younger adults [2]. Hispanic or Latino populations in the United States are disproportionately impacted, having significantly higher rates of type 2 diabetes adjusted for age and sex (22.1%) relative to non-Hispanic racial and ethnic groups (ie, 12.1%, 19.1%, 20.4%, 18.5% for White, Asian, Black, and other race, respectively) [3]. Hispanic or Latino individuals also tend to have worse type 2 diabetes outcomes, including poorer glycemic control and higher rates of diabetes-related complications [4-6]. Notably, the Hispanic or Latino population is one of the largest and most rapidly growing racial and ethnic minority groups in the United States [7] and in Utah [8] where the current project is based, highlighting the need to improve health outcomes in this population.

Lifestyle interventions, which focus on changing health behaviors (eg, increasing moderate-to-vigorous physical activity [MVPA] and improving nutrition), have shown efficacy in successfully preventing or delaying type 2 diabetes across racial and ethnic groups, including Hispanic or Latino individuals [9]. Despite this, when lifestyle interventions are translated to community and health care settings (most notably, the National Diabetes Prevention Program [National DPP]), Hispanic or Latino participant outcomes are poorer than those of other racial and ethnic groups [10]. This disparity is largely explained by low attendance rates, as each additional intervention session attended is associated with an additional 0.31% weight loss [10]. Hispanic or Latino participants in the National DPP had lower attendance and retention rates, with only 52.6% of Hispanic or Latino participants being retained through the 18th week of the year-long intervention, compared to 70.5% for non-Hispanic or Latino White individuals [11]. Hispanic or Latino participants were also underrepresented among National DPP participants (only 10% of enrolled participants were Hispanic or Latino) [10], a result that is particularly concerning given the high prevalence of type 2 diabetes in this group [3]. Thus, despite the disproportionate impact of type 2 diabetes on Hispanic or Latino individuals, large-scale real-world translations of efficacious lifestyle interventions for diabetes prevention have not effectively enrolled and retained Hispanic or Latino individuals.

Further research to explain this pattern is sparse. Disparities in engagement might be related to cultural incongruence between aspects of the lifestyle intervention program curriculum in addition to previously reported structural barriers. Critically, qualitative evidence suggests the typical length of lifestyle interventions for diabetes prevention (eg, 22 classes over the course of 1 year in the National DPP) is a barrier to participation and completion of the program for Hispanic participants [12,13]. Another key barrier cited by Hispanic participants in the National DPP is the lack of family support [14].

There is substantial evidence that the health and lifestyles of family members tend to be concordant [15]. While research has increasingly recognized and emphasized the importance of the social context of an individual in influencing lifestyle change, diabetes prevention interventions have not systematically integrated family members into programs. A family-based approach may be beneficial for Hispanic or Latino participants given the core Hispanic or Latino cultural value of familism, which emphasizes the family unit [16]. A family-based approach may facilitate increased family support, which is needed given that lack of family support was a top barrier identified among Hispanic or Latino National DPP participants [14]. There is preliminary work to support this idea. For example, in a secondary analysis of participant engagement in a predominantly Hispanic or Latino-serving National DPP, participants had higher rates of retention when participating with someone in their household, and Hispanic or Latino participants were more likely to participate with a household member relative to non-Hispanic or Latino participants [17]. Further, in a culturally adapted lifestyle intervention for individuals with type 2 diabetes or at high risk for the disease, Hispanic or Latino participants who participated with at least one family member or friend had, on average, statistically significant weight loss by the end of the program, whereas Hispanic or Latino participants who participated alone did not [14]. Qualitative results illustrated that participants found support and attendance from family members crucial to their success in the program. This work provides preliminary support for incorporating family members into the National DPP, as participating with a family member might improve engagement and outcomes in Hispanic or Latino individuals.

Culturally adapting lifestyle interventions for diabetes prevention is an increasingly supported avenue for improving engagement in minoritized communities [18]. In the National DPP specifically, Hispanic or Latino participants had higher rates and longer lengths of attendance when they enrolled in programs that used cultural adaptations to tailor the program to specific community needs [19]. Thus, while lifestyle interventions hold promise for preventing or delaying type 2 diabetes in Hispanic or Latino individuals, specific adaptions may facilitate more effective engagement and retention in these individuals. In the current project, we focused on decreasing the length of the program and systematically incorporating family members into the intervention.

In the context of a larger community-based participatory research project, we developed PrevenganT2, a family-based lifestyle intervention designed to meet the needs of Hispanic or Latino adults in Utah. The larger project, Adapting Diabetes Interventions to Improve Outcomes and Stop Type 2 Diabetes in Hispanic or Latino Communities (ADIOS T2D!) [20], is guided by the Obesity-Related Behavioral Intervention Trials (ORBIT) framework for early-phase intervention development [21,22]. Described in more detail below, the adaptation (ie, ORBIT phase 1b, “refine” phase) was based on data collected from Hispanic or Latino family dyads in a formative evaluation trial of the core curriculum (ie, first 16 classes) of the 2021 version of the Centers for Disease Control and Prevention (CDC) Spanish-language PrevengaT2 curriculum. Specific changes were recommended by members of a Community Advisory Board (CAB) who are coauthors of this manuscript.

Here, we describe the protocol for a proof-of-concept study. In the ORBIT model, a proof-of-concept trial is one of 3 approaches to preliminary testing of a behavioral intervention. The primary goal of a proof-of-concept (ORBIT phase 2a) study is to evaluate whether the intervention alters a treatment target in a clinically significant way (ie, whether there is a “clinically significant signal” of PrevenganT2 on MVPA, the primary outcome and treatment target of focus) in Hispanic or Latino adults at high risk for type 2 diabetes in a community setting in Utah. Consistent with ORBIT recommendations for this subphase of “preliminary testing,” the design was nonrandomized and included a small sample of participants in each of 2 counties [23]. If warranted, additional preliminary testing trials will evaluate feasibility and acceptability, including recruitment and retention (ie, phase 2b; feasibility pilot trial) as well as efficacy (ie, phase 2c; phase II efficacy trial) [23]. The results of this trial will determine our next steps in this intervention development work to better meet the needs of Hispanic or Latino communities. If the intervention meets prespecified criteria for a “clinically significant signal” on MVPA (Data Analysis), additional preliminary testing will follow. In contrast, if the intervention does not meet the prespecified criteria, additional ORBIT phase 1 work (ie, refinement) will be carried out instead.

## Methods

### PrevenganT2 Development

**Figure 1:**
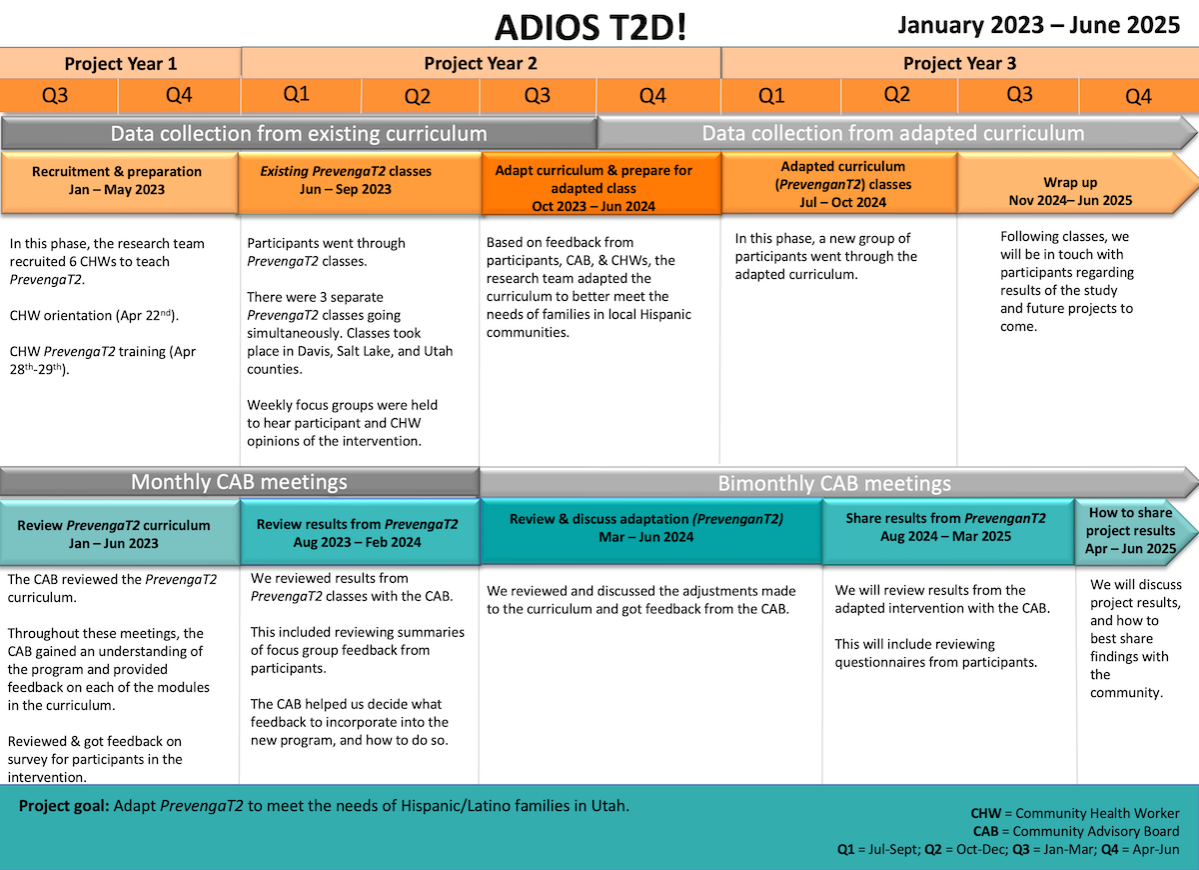
Overall project timelines. ADIOS T2D!: Adapting Diabetes Interventions to Improve Outcomes and Stop Type 2 Diabetes in Hispanic or Latino Communities.

For a visual overview of the larger ADIOS T2D project, refer to Figure 1. We used an iterative approach to intervention development guided by the ORBIT framework and members of the Hispanic or Latino community. A 7-member CAB formed in January 2023 included individuals with personal or professional experience with diabetes prevention in the Hispanic or Latino community. Members included health care providers, community health workers (CHWs) with prediabetes experience, people with prediabetes, family members of someone with prediabetes, and community members.

First, the CAB reviewed the existing PrevengaT2 core curriculum and provided initial feedback. This feedback informed focus group questions for participants in a formative evaluation trial. Additional details about the formative evaluation trial methods and results are available from the study authors. Immediately following each of the 16 modules delivered weekly by CHWs in the formative evaluation, separate focus groups were held for target participants (ie, individuals at high risk for type 2 diabetes) and family members (ie, adult family members living in the same household as the target participant). The team also conducted brief CHW debrief meetings following each module. Following qualitative coding of focus groups and CHW debrief meetings, the research team presented deidentified results to the CAB. The CAB provided guidance to the research team on how feedback from participants and CHWs could be implemented in the adaptations of the PrevengaT2 core curriculum.

The CAB members recommended 14 weekly classes, lasting 1 hour each, and changing the language of the curriculum to include a warmer and more inviting tone as well as inclusive discussion of family members. Visuals were enhanced to incorporate more vibrant colors and culturally relevant imagery that resonates with Hispanic or Latino participants, such as food and family-oriented images. The curriculum incorporated links to Spanish-language resources on exercises, recipes, and mental health to address requests for more comprehensive information. Finally, the focus on weight was deemphasized to more effectively promote health behaviors such as balanced nutrition and physical activity. Weight-centric messaging in PrevengaT2 was not viewed as motivating by participants and was upsetting for some.

### PrevenganT2 Location/Setting

This intervention took place in Utah. The project is a collaboration between researchers at the University of Utah and a local Hispanic or Latino-serving community-based organization, Alliance Community Services. A total of 2 cohorts of dyads (one in each of 2 local counties) participated in the intervention.

### Recruitment, Eligibility, and Participants

Recruitment occurred in June and July of 2024. CHWs recruited participants through in-person contact, phone calls, and outreach to community members who previously agreed to future contact from Alliance Community Services during a project focused on diabetes screening. CHWs aimed to recruit 10-12 target participants along with an adult family member (n=20-24) across 2 counties (ie. 5-6 family dyads per county). Given the emphasis in an ORBIT proof-of-concept trial on examining whether there is a clinically significant change in behavior under ideal conditions [23,24], our sample size was based on what we thought would be the ideal number of participants in each of the 2 groups based on the previous experience of our team. Specifically, we delivered the CDC National DPP curriculum to dyads in the intervention refinement phase of the larger study, members of the team have been involved with intervention delivery to romantic partner dyads in couple-based work [25], and Alliance Community Services team members have delivered interventions in the Hispanic or Latino community for decades. Based on these experiences, we determined 5-6 dyads to be large enough to encourage discussion among members while small enough to maintain a focus on the specific needs of the individuals in the room.

To be eligible for participation in the study, target participants were required to be at least 18 years old, identify as Hispanic or Latino, be fluent in English or Spanish, and be at high risk for type 2 diabetes based on the American Diabetes Association/CDC diabetes risk test [26,27]. Target participants were also required to have a family member living in their home willing to participate in the study with them. Inclusion criteria for family members were being at least 18 years old and fluent in Spanish or English. Exclusion criteria for targets and family members included having type 1 or type 2 diabetes, current medication for diabetes, current enrollment in a lifestyle intervention for prediabetes or obesity, and being a CAB member in this study. Trained and experienced CHWs (n=2) delivered PrevenganT2 in each of the 2 counties (Salt Lake County, Davis County).

### Intervention Procedures

Participants (ie, both targets and family members) attended up to 14 weekly PrevenganT2 intervention classes, each lasting 60 minutes. Each class focuses on a different module from the adapted curriculum, including physical activity, nutrition, tracking physical activity and nutrition, and stress management. The intervention was delivered in Spanish. In addition to compensating participants for their time completing study assessments, participants received incentives at each class. CHWs chose incentives related to the topic of a given class. If needed, participants were given vouchers for childcare and transportation to and from the classes.

### Assessment Procedures, Outcomes, Measures, and Materials

#### Assessment Procedures

Before beginning the intervention (pre), participants completed consent forms and filled out a series of questionnaires in Spanish. In addition, target participants engaged in a 7-day assessment during which they wore ActiGraph GT3X-BT accelerometers on their waists to measure MVPA. Participants were instructed to wear accelerometers 24 hours/day around their waist and were sent a link to an internet-based questionnaire each morning to validate the accelerometer data. These procedures were repeated after the final intervention class (post).

#### Measures and Materials

We selected target partner MVPA as the primary outcome of this study. Although other health behaviors are targeted in the National DPP and measured as secondary outcomes (eg, sleep), the emphasis in the CDC intervention is primarily on increasing MVPA and improving nutrition. As increases in MVPA affect cardiovascular risk [28], and MVPA was the primary behavioral outcome in both the initial efficacy trial [9] and the National DPP evaluation [10], this was selected as the primary behavioral outcome in this proof-of-concept trial. We use accelerometry to assess MVPA. Objective approaches are recommended for precise measurement of physical activity in the context of lifestyle interventions [29,30]. Accelerometry is the most common objective approach for physical activity measurement in daily life [31] and the preferred tool for assessing the effects of interventions on physical activity [32]. As described above, target participants were fitted with waist-worn Actigraph GT3X-BT devices that they were instructed to wear for 7 consecutive days before (pre) and after (post) the intervention. To be considered a valid week of objective MVPA data, participants must have had at least 4 valid days of data (including at least one weekend day). A valid day of data is defined as at least 10 hours of wear time while awake [32,33]. The Freedson Adult VM3 2011 algorithm, along with sedentary cut points based on previous recommendations [34] will be used to calculate MVPA, operationalized as the total minutes at 2690 counts per minute and higher on valid days [34,35]. For participants with a valid week of data, MVPA minutes over the course of the week will be determined by multiplying the average MVPA minutes across all valid days by 7 days.

To contextualize the sample, self-report measures in the prequestionnaire included demographic information, the American Diabetes Association/US Centers for Disease Control and Prevention Prediabetes Screening Test [26,27], and cultural factors (ie, acculturation, measured by the Short Acculturation Scale for Hispanics [SASH)] [36] and familism, measured by the Short Attitudinal Familism Scale [SAFS]) [37]. Although the emphasis in a proof-of-concept trial is on whether there is a change in a specific behavioral target [23], we also collected secondary outcome data from participants, including measures of health behaviors (ie, nutrition, measured by the Latino Dietary Behaviors Questionnaire [LDBQ] [38] and physical activity, measured by the International Physical Activity Questionnaire [IPAQ]) [39], mental health (ie, symptoms of anxiety, measured by the Generalized Anxiety Disorder-7 Scale [GAD-7] [40], and depression, measured by the Patient Health Questionnaire [PHQ-8]) [41], and social support from participating family members (ie, for diet, measured with the Social Support for Diet scale [SSDS], and exercise, measured with the Social Support for Exercise scale [SSES]) [42]. In addition, CHWs recorded program engagement (ie, attendance) at each class; participant weight was measured with a medical-grade scale at classes 0, 4, 8, and 13; and participant-reported MVPA minutes from the previous week were reported at each of classes 7-13.

When possible, we used measures developed in Spanish or Spanish translations of measures subject to psychometric evaluation among Latino participants. These measures include the SAFS [37], International Physical Activity Questionnaire [43], SASH [36], PHQ-8 [44], GAD-7 [45], SSDS [46], and SSES [46]. For measures that lacked culturally appropriate translations (ie, for measures not originally developed for use in Spanish or had not been validated in Latino samples), English measures were translated by a team of certified translators and subsequently reviewed by the CAB prior to use. In addition, minor edits were made to the wording of questions and responses for clarity, simplicity, and grammar on the SSES, GAD-7, PHQ-8, SASH, and LDBQ. For example, we made edits to the SASH [36], for proper punctuation (ie, “que” to “qué”). Edits were also made to the language of secondary measures to incorporate the family-based approach. The language was changed from a singular participant to the family members participating in the program with them as they are going through the program as a unit (ie, “usted” to “ustedes”). This approach of translation was not validated but was changed to fit the needs that the participants suggested. Changes were also made to the tone of the language to be more inviting and engaging. Information regarding all specific edits made to questionnaires is available from the corresponding author upon request.

### Planned Data Analysis

A clinically significant increase in MVPA among target participants will be calculated using pre and post-accelerometer data and will be defined as either (1) meeting the recommendation of MVPA ≥ 150 minutes per week at post (among those not meeting it at pre), or (b) a pre-to-post increase ≥ 35 MVPA minutes per week. These criteria are based on current adult physical activity guidelines in the United States [47] as well as the finding that the equivalent of 5-6 minutes per day of brisk walking is the “minimum clinically important difference” in physical activity to reduce cardiovascular risk among inactive adults [28]. Although we took steps to collect a valid week of accelerometer data, some target participants may not have a valid week of accelerometer data in pre or post. Target participants with invalid data at one or both time points may meet the criteria for clinically significant increase in MVPA based on subjective measures (ie, IPAQ or self-reported MVPA recorded at classes). Although subjective assessment of physical activity is not considered the gold standard method of assessment, the efficacy trial and National DPP evaluation used MVPA self-reports [9,10], and our team determined the costs of the potential loss of data to outweigh the costs of using subjective methods in a small number of cases.

We will report the percentage of target participants demonstrating a clinically significant increase in MVPA, descriptive statistics of sociodemographic characteristics at pre, and descriptive statistics of all outcomes at pre and post. While we do not have the power to evaluate pre-post changes with inferential statistics, we will observe patterns in secondary outcomes to contextualize findings and inform our next steps.

### Ethical Considerations

All participants provided informed consent. This study protocol was approved by the University of Utah institutional review board (#0017800) on June 18, 2024. Each participant provided written informed consent in Spanish prior to beginning the study. As part of the consent process, participants were given the option to consent to or opt out of data sharing. All data will be deidentified following data collection. Participants were compensated up to US $200 in gift cards for completing study assessments.

## Results

This trial was in progress at the time of the initial submission of this paper. Data collection concluded in October 2024 and data cleaning and preparation for analysis are ongoing.

## Discussion

### Overview

The results of this proof-of-concept trial of PrevenganT2, a culturally adapted lifestyle intervention, will inform our team’s next steps. We expect the majority of target participants will illustrate clinically significant increases in MVPA from preintervention to postintervention. If this is the case, our team will proceed with additional preliminary testing to determine the feasibility and acceptability of PrevenganT2 and a randomized study protocol (ORBIT phase 2b), as well as the initial efficacy of PrevenganT2 (ORBIT phase 2c), before carrying out a larger randomized trial. If the majority of target participants do not demonstrate clinically significant pre-post increases in MVPA, we will carry out additional intervention refinement (ie, ORBIT phase 1b). As noted above, we do not have the power to evaluate pre-post changes with inferential statistics, but will contextualize our findings by observing patterns of change that inform our next steps. The results of this study will be disseminated through publication in a peer-reviewed journal and presentation at local and national conferences. In addition, results will be disseminated directly into the community in meetings with the CAB and with our community partners at Alliance Community Services.

### Comparison to Prior Work

As a whole, individually focused lifestyle interventions to prevent type 2 diabetes have failed to effectively engage Hispanic or Latino individuals and have not systematically integrated family members into interventions. In recent years, there has been increased recognition of the need to develop culturally responsive lifestyle interventions to address the disproportionate burden of type 2 diabetes in Hispanic or Latino communities. In turn, a number of such interventions have been developed and some illustrated the potential efficacy of culturally tailored lifestyle interventions in reducing the risk of type 2 diabetes through relevant outcomes like a clinically significant reduction in hemoglobin A_1c_ [48] and weight [48,49] in Hispanic or Latino communities. However, these studies have varied substantially in factors such as the level of cultural adaptation (eg, many have simply translated the program from English to Spanish), the extent of community engagement, and outcomes assessed (eg, looking exclusively at weight or hemoglobin A_1c_). Further, evidence for the impact of these prior interventions on physical activity and other behavioral outcomes is weak, and to our knowledge, no studies have specifically examined changes in MVPA [50]. Moreover, none of these studies have systematically included family members in the lifestyle intervention and most have methodological limitations. The results of this study will be interpreted in the context of these limitations to prior work.

### Strengths and Limitations

This project has a number of strengths. Most notably, it was designed and carried out in close collaboration with members of local Hispanic or Latino communities. Our strong community partnerships and formation of a CAB to guide the larger project allowed us to adapt the intervention to the specific needs of local community members. Further, hiring CHWs from the Hispanic-serving organization, we partnered with facilitates capacity building within the organization and in turn, the potential sustainability of the intervention. Another strength of this project is the use of the ORBIT model to guide this early intervention development work. This model will guide us in refining and strengthening aspects of the intervention and study protocol if needed before scaling up to a larger trial that requires substantially more resources. Another strength is our objective measurement of physical activity. The use of accelerometers minimizes much of the bias introduced by self-report measures of physical activity.

Despite these strengths, this project also has limitations. Although we consider our community-based approach and use of the ORBIT model a strength, the primary focus in proof-of-concept trials is on whether there is a “clinical signal” on a primary behavioral outcome under ideal conditions [23,24]. Despite the strong scientific rationale for our focus on MVPA as the primary outcome, our experience with the broader ADIOS T2D! project suggests that MVPA may not be the most culturally relevant outcome for Hispanic or Latino communities. As noted above, MVPA was not a behavioral outcome in previous evaluations of diabetes prevention work in Hispanic or Latino communities. Inadequate representation of Hispanic or Latino participants in much of the science on which our primary outcome rationale was based is a clear limitation that in hindsight should have been more carefully considered together with the CAB. Whereas community engagement is in no way inconsistent with the ORBIT model, the field would benefit from recommendations for how to optimally engage communities in intervention development guided by the ORBIT model. Based on our experience, we recommend researchers explicitly discuss with community members the most relevant behavioral outcomes in their community when selecting primary and secondary outcomes. Further, we recommend researchers working with minoritized communities critically evaluate the extent to which the community has been represented in the relevant research literature. In the meantime, the narrow focus of this trial, along with the small sample size and nonrandomized design, limit the potential conclusions we can draw. Feasibility outcomes including attendance of target and family member participants will be formally evaluated in future preliminary testing (ie, in an OBBIT phase 2b feasibility trial). In addition, the program is limited to individuals with a family member who is willing and able to participate in the intervention. Although we carefully considered how to increase access to the program by limiting known barriers to lifestyle intervention engagement (eg, offering participants childcare and transportation vouchers and use of incentives), the requirement of family member participation may restrict intervention access to those with more social resources.

### Conclusions

Type 2 diabetes preventive interventions that meet the needs of Hispanic or Latino communities are critical and have the potential to substantially improve health outcomes in the Hispanic or Latino population. This study addresses this through close partnerships with community members and by leveraging family relationships. Although the conclusions we can draw from this small trial are limited, they will inform the next steps of our team and others. This project represents the next step in our understanding of the potential for family-based lifestyle interventions to increase engagement, improve outcomes, and increase health equity in the Hispanic or Latino community.

## Appendix

Multimedia Appendix 1 Peer review reports by the American Diabetes Association Research Grant Review Committee.

## Abbreviations

ADIOS T2D!: Adapting Diabetes Interventions to Improve Outcomes and Stop Type 2 Diabetes in Hispanic or Latino Communities
CAB: Community Advisory Board
CDC: Centers for Disease Control and Prevention
CHW: community health worker
DPP: Diabetes Prevention Program
GAD-7: Generalized Anxiety Disorder-7 Scale
IPAQ: International Physical Activity Questionnaire
LDBQ: Latino Dietary Behaviors Questionnaire
MVPA: moderate-to-vigorous physical activity
ORBIT: Obesity-Related Behavioral Intervention Trials
PHQ-8: Patient Health Questionnaire
SAFS: Short Attitudinal Familism Scale
SASH: Short Acculturation Scale for Hispanics
SSDS: Social Support for Diet scale
SSES: Social Support for Exercise scale

## Data Availability

The datasets generated during this study are not publicly available to protect the identities and information of participants from vulnerable populations. Data from participants who consented to data sharing are available from the corresponding author pending agreement to conditions of data access.
