# Supplementary material for: “PrevenganT2,” a Culturally Responsive Family-Based Diabetes Prevention Intervention for Hispanic or Latino Adults at High Risk for Type 2 Diabetes: Protocol for a Proof-of-Concept Evaluation
Source: JMIR Res Protoc. 2025 Aug 14;14:e66317. doi: 10.2196/66317 (PMC12395107; doi:10.2196/66317)
Supplement: Multimedia Appendix 1 [file resprot_v14i1e66317_app1.pdf]

ADA Grant Application: Reviewer Critiques

Grantquestions <Grantquestions@diabetes.org>
Mon 6/6/2022 1:16 PM
To: Katie Baucom (she/her/hers) <katherine.baucom@psych.utah.edu>

Dear Dr. Baucom,

Thank you for your recent grant application to the American Diabetes Association. As one of ADA's grant review best practices and to ensure full transparency, we are providing you with the external reviewer comments below related to your submission, Grant ID# 7-22-ICTSN-36, titled, "Family-based lifestyle intervention to prevent diabetes in Hispanic/Latino communities in Utah".

On behalf of the American Diabetes Association, thank you for your efforts to advance our mission to prevent and cure diabetes and to improve the lives of all people affected by diabetes. Should you have questions, please don't hesitate to contact Association staff at grantquestions@diabetes.org.

Reviewer 1 Feedback

What is the overall hypothesis the proposal seeks to address?
The investigators in this proposal hypothesize that the existing diabetes prevention program has only had modest effectiveness with individuals of Hispanic ethnicity because cultural adaptations have not been made to make the program more relevant. This is related to the individually-focused approach to behavior change. They posit that the program has not systematically included family members which would appeal to the familial values of people of Hispanic ethnicity. In addition, Hispanic participants may find the structure and duration of the NDPP (22+ classes in 12 months) too burdensome. They therefore propose to adapt the intervention for culturally responsive family-based delivery and increase ts efficiency.

What are the major strengths and weaknesses of the proposal?
This well-written proposal clearly lays out the need for cultural adaptation and there is a clear, community engaged plan to complete the work. The study team has been conducting relevant work in this community and has strong connections that will help the investigators complete their aims AND obtain meaningful, impactful feedback. I appreciate the investigators' skill in orienting this work within the ORBIT model which helps to justify their methods, particularly in Aim 1. The proposal is appropriately placed in the formative stage which is a significant strength. A minor weakness to consider is that the investigators do not lay out a concise plan for how adaptations identified in Aim 1 will be integrated into Aim 2. As written, it seems the investigators assume the main adaptations needed will be to written materials. However it is not very likely these alone will address the main barriers to participation and engagement.

How could this proposed research be improved? Please be specific.
I have some minor suggestions for strengthening the proposal, but these do not significantly decrease the scientific merit of the proposal. First, I expect that the feedback and suggestions from the CAB and the participants in Aim 1 will yield some important modifications. More detail on how this will be integrated into Aim 2 would be helpful. The investigators suggest that they have a consultant in place to help with editing but it is not clear what will be edited--just materials? If the number of sessions, the in person nature of the sessions, time, etc. are all challenges for participation, as the investigators suggested, then these will surely need to be addressed in addition to the written materials. In fact, the supposition that the written materials will need to be edited assumes that participants will find them helpful, which may or may not be the case. If the primary outcome is PA then it may be that written materials are not very informative. Another issue to consider is eligibility in terms of PA if participants are already sufficiently active, is it reasonable to expect an increase? Or that this will yield a meaningful change in biological outcomes? Finally, the investigators suggest that a COVID pivot for Aim 2 would be to move to virtual classes. It is possible, however, that virtual classes

may be a reasonable adaptation identified in Aim 1.

Is the proposed research being addressed by others?
This is an innovative proposal with an appropriate study design for the work proposed.

If successful, what would be the potential impact of the proposed research? Ex: how will this improve lives of patients living with diabetes?
Given that people of Hispanic ethnicity are at higher risk for DM, this is a timely proposal that will address a critical gap in our current knowledge of behavioral treatment.

Will the experimental approaches/proposed methods allow for a clear conclusion?
Yes. Based on the outlined proposal, the investigators will likely receive substantive feedback and recommendations for program adaptation. Their findings will be translatable directly to the existing DPP programs.

Can this work be accomplished within the allocated time frame?
No concerns.

Is the proposed research innovative or incremental?
This is an innovative proposal that will fill gaps in our current knowledge for making DPP relevant to a population that is in great need of further attention.

Does the applicant have appropriate qualifications to perform the work?
This is a strong team with previous collaborations.

Are appropriate resources available to the investigator to perform the proposed research (ex: environment, facilities, equipment)?
No concerns.

Do you have any ethical concerns about the proposed study? If yes, please describe your concerns in detail. If no, please enter N/A.
No Concerns.

Reviewer 2 Feedback

What is the overall hypothesis the proposal seeks to address?
A less burdensome, culturally-responsive, family-based intervention for Hispanic adults at risk for type 2 diabetes will effectively reach and engage participants, and facilitate lifestyle change.

What are the major strengths and weaknesses of the proposal?
Strengths: Excellent research question and hypothesis
Weaknesses: The proposal and methodology will not really address the hypothesis and further work on clinical endpoints is needed.

How could this proposed research be improved? Please be specific.
Further refine protocol to include larger numbers, longer follow up and clinical endpoints.

Is the proposed research being addressed by others?
No

If successful, what would be the potential impact of the proposed research? Ex: how will this improve lives of patients living with diabetes?
If successful, and I don't believe the proposal as it stands would lead to this - but in theory addressing

hypothesis would lead to diabetes remission, less complications.

Will the experimental approaches/proposed methods allow for a clear conclusion?
No

Can this work be accomplished within the allocated time frame?
Yes

Is the proposed research innovative or incremental?
This is an innovative proposal that will fill gaps in our current knowledge for making DPP relevant to a population that is in great need of further attention.

Does the applicant have appropriate qualifications to perform the work?
Yes

Are appropriate resources available to the investigator to perform the proposed research (ex: environment, facilities, equipment)?
Yes

Do you have any ethical concerns about the proposed study? If yes, please describe your concerns in detail. If no, please enter N/A.
N/A

Reviewer 3 Feedback

What is the overall hypothesis the proposal seeks to address?
Since large-scale translations of lifestyle interventions from efficacy trials to community settings have not effectively reached or engaged Hispanic/Latino adults, a less burdensome, culturally-responsive, family-based intervention for Hispanic adults at risk for type 2 diabetes will effectively reach and engage participants, and facilitate lifestyle change.

What are the major strengths and weaknesses of the proposal?
Strengths:
-Engagement of a Community Advisory Board to adapt the Diabetes Prevention Program, by developing a culturally-responsive, family-based diabetes prevention program, for Hispanic adults at risk for type 2 diabetes
-Adjustment to previous efforts based on findings that existing interventions are too burdensome
-Research focused on Hispanic/Latino adults, a group having the highest prevalence of type 2 diabetes of all racial/ethnic groups in the US
-Plan to produce a scalable lifestyle intervention for Hispanic adults in Utah at risk for type 2 diabetes that could be utilized in similar populations nationwide
-Inclusion of family members, who may have diabetes or be at risk for diabetes, in the intervention
-Research team with members who are bilingual and knowledgeable about the Hispanic/Latino cultural
Weaknesses:
-Lack of weight data and specific behavioral changes related to nutrition which may give useful information in this study and guide future research

How could this proposed research be improved? Please be specific.
-The main outcome in the proof-of-concept evaluation (Aim 2) will be increase in moderate to vigorous physical activity with an objective measure (wear of an activity monitor) and a subjective measure (activity logs). Nutrition data will be collected for secondary analyses. There is a rationale for not including more dietary information or weight (increased physical activity, even without weight loss, can improve glycemic control) but the addition of weight measurements could provide information (whether there is a change or not).

Is the proposed research being addressed by others?
There does not seem to be duplicative research in this particular group with the methodology described.

If successful, what would be the potential impact of the proposed research? Ex: how will this improve lives of patients living with diabetes?
The main goal is to improve diabetes prevention efforts in Hispanic/Latino communities through the implementation of a culturally-responsive, family-based program. This is a group that has very high rates of prediabetes and type 2 diabetes. The intervention can not only help the individual at risk for prediabetes and diabetes but also other family members who are likely at risk as well.

Will the experimental approaches/proposed methods allow for a clear conclusion?
Yes, the two major aspects of the experimental methods (Advisory Board activities and the Diabetes Prevention Program) will allow for clear conclusions regarding the feasibility of the adapted DPP for this population. Further research will be needed, and is proposed, to capture data on other aspects of the DPP (change in progression from prediabetes to type 2 diabetes, weight change, etc.).

Can this work be accomplished within the allocated time frame?
Yes, the timeframe seems reasonable for completion of all aspects of the work.

Is the proposed research innovative or incremental?
The proposed research is incremental, building on the work of the National Diabetes Prevention Program (NDPP), with the goal of submitting an NIH R01 proposal for a larger trial. Previous work has demonstrated benefit from inclusion of another household member in the Hispanic NDPP. This proposal builds on this with adjustments to address other barriers (NDPP is too burdensome/too time-consuming for Hispanic participants). The applicant has published on the promises and pitfalls of dyads in the NDPP and barriers to participation and lifestyle change among lower vs. higher income participants in the NDPP which lays some of the groundwork for this research.

Does the applicant have appropriate qualifications to perform the work?
Yes, this applicant has the necessary background and research history to perform the work. The applicant has created a team to support the research.

Are appropriate resources available to the investigator to perform the proposed research (ex: environment, facilities, equipment)?
Yes, there are appropriate resources to perform the proposed research including a team of researchers, environment to carry out the research (including access to those who will participate), facilities and equipment needed.

Do you have any ethical concerns about the proposed study? If yes, please describe your concerns in detail. If no, please enter N/A.
N/A
